# Synthesis and gas sensing properties of membrane template-grown hollow ZnO nanowires

**DOI:** 10.1186/s40580-017-0121-2

**Published:** 2017-10-25

**Authors:** Jae-Hyoung Lee, Jin-Young Kim, Jae-Hun Kim, Ali Mirzaei, Hyoun Woo Kim, Sang Sub Kim

**Affiliations:** 1 Department of Materials Science and Engineering, Inha University, Incheon, 22212 Republic of Korea; 2 Division of Materials Science and Engineering, Hanyang University, Seoul, 04763 Republic of Korea

**Keywords:** Hollow, ZnO, Nanowire, Membrane, Surface area, Gas sensor

## Abstract

One-dimensional, hollow nanostructured materials are among the most promising materials for sensing applications owing to their high surface area that facilitates the adsorption of target gases. Accordingly, for gas sensing studies, hollow ZnO nanowires (NWs) with different surface areas were successfully synthesized herein by using polycarbonate membranes with different pore sizes as templates, and deposition of ZnO via the atomic layer deposition technique. The sensing properties of the synthesized hollow ZnO NWs were examined for CO and NO_2_, revealing their comparative sensing performances with ZnO nanomaterials-based sensors reported in literature. This study highlights a novel way of synthesizing hollow ZnO NWs by using membrane template and their promising sensing properties as well.

## Introduction

Because of increasing concerns about air pollution, public security, and the high standards of modern life, gas detection has gained increasing importance [1, 2]. Metal-oxide gas sensors are most commonly used for the detection of ambient gases based on their high response, fast and dynamic characteristics, easy fabrication, portability, and cheapness [3]. However, the performance of these sensors must be enhanced to meet the demands of the high standards of living. One promising approach for enhancing the gas-detection capability of metal-oxide-based gas sensors is to increase the surface area of the sensor [4]. In fact, a higher surface area generally results in greater availability of sites for gas adsorption, and accordingly, higher performance. Many researchers have investigated high-surface-area metal-oxides such as nanofibers (NFs) [5], nanorods [6], nanowires (NWs) [7], and hierarchical [8] and porous materials [9] for gas sensing applications. In order to further increase the surface areas of such nanomaterials, hollow nanostructured nanomaterials can be employed. Such morphologies offer more adsorption sites as they possess inner and outer surfaces, meaning that the surface-to-volume ratio almost doubles compared with that of the normal solid counterparts; therefore, higher sensing performance is expected [10].

In our previous work [11], we fabricated hollow ZnO NFs with different diameters via the electrospinning method. It was found that ZnO NFs with smaller diameters were more sensitive to both reducing and oxidizing gases than those with larger diameters. In another study [12], we found that the sensing performance of ZnO hollow NFs depended on their wall thickness, where the ZnO hollow NFs with thinner walls showed better sensing performance. More recently [13], we reported TiO_2_/ZnO inner/outer double-layer hollow NFs that exhibited sensitive and selective detection of reducing gases. Zhang et al. [14] compared the CO gas sensing properties of hollow and normal TiO_2_ NFs; the hollow TiO_2_ NFs showed better sensing performance because of the effect of the increased surface-to-volume ratio derived from generation of the inner surfaces. Park et al. [10] reported that a hollow ZnO NFs sensor showed much higher sensitivity to NO_2_, when compared to normal ZnO NFs, owing to the increased surface area of the former.

In most of these literature studies on hollow nanostructures, the focus was placed on hollow NFs and less attention has been paid to other hollow nanostructures such as hollow NWs. NW gas sensors exhibit many inspiring characteristics such as (i) ultra-sensitivity and fast response time, (ii) higher selectivity and stability, (iii) light weight, (iv) low power consumption, and (v) wireless communication applicability [15]. Therefore, it is of importance to increase the performance of NW gas sensors by increasing the surface area through the fabrication of hollow NWs. Accordingly, in this work, we report the novel synthesis, characterization, and sensing performance of hollow ZnO NWs prepared using cyclopore polycarbonate membranes (with different pore sizes) as templates with subsequent deposition of ZnO via atomic layer deposition (ALD). The membrane templates were removed by combustion at 450 °C over 4 h. Scanning electron microscope (SEM) images demonstrate formation of the hollow ZnO NWs. Gas sensing tests towards CO and NO_2_ gases reveal the higher performance of the gas sensors with higher surface area. The sensing mechanism is also discussed in detail.

## Experiment

### Synthesis of hollow ZnO NWs

**Fig. 1:**
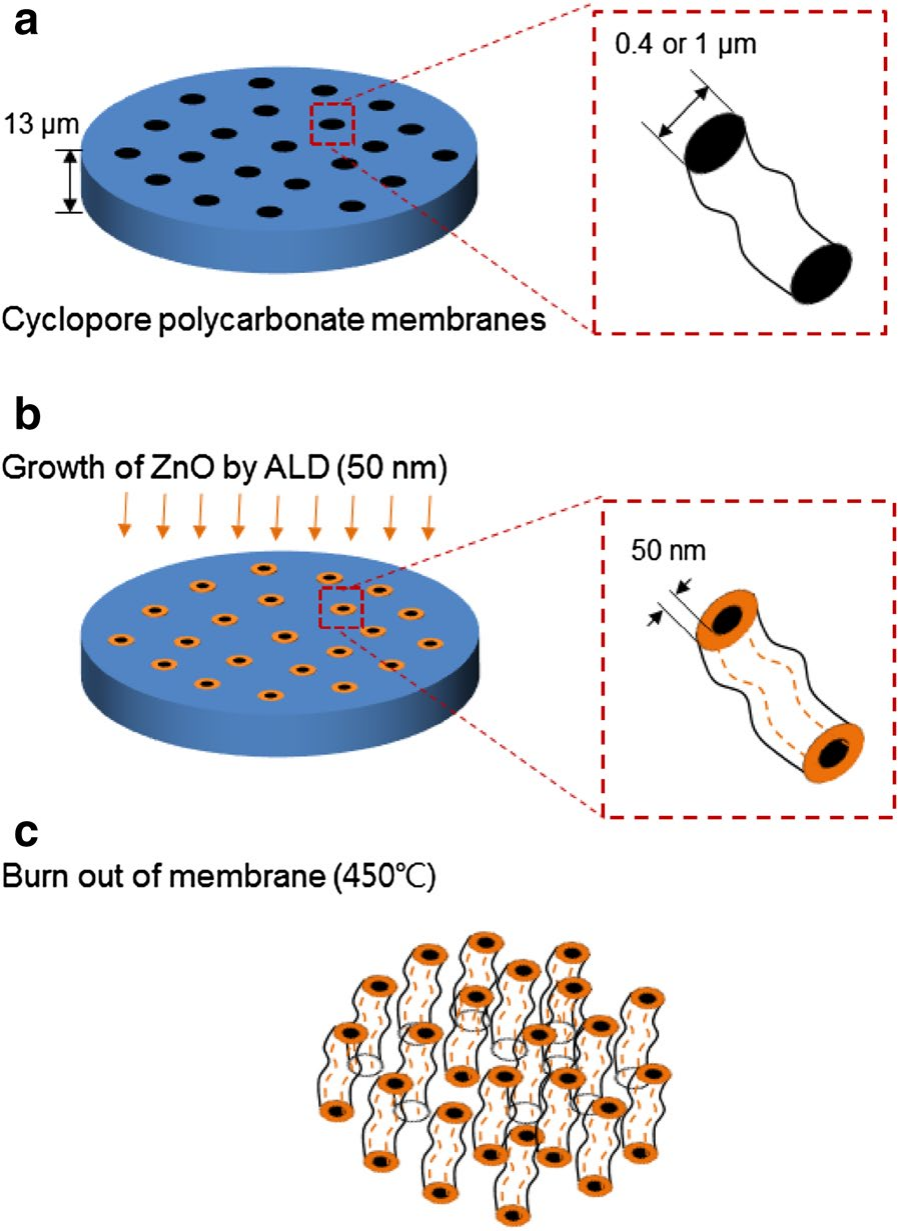
Schematic illustration of steps used for the preparation of hollow ZnO NWs. **a** Cyclopore polycarbonate membranes. **b** Growth of ZnO by ALD (50 nm). **c** Burn out of membrane (450 °C)

The hollow ZnO NWs were prepared on SiO_2_ (200 nm thick)-grown Si (100) substrates using the membrane-template method and ALD technique. For synthesis of the hollow ZnO NWs, cyclopore polycarbonate membranes (Whatman) were used as templates. The two membrane-templates had a diameter of 25 mm and thickness of 13 μm, and respective pore diameters of 0.4 and 1 μm. ZnO was deposited on these membranes via a conventional ALD technique, as described in our previous paper [16]. ALD was performed by sequential exposure of the cyclopore polycarbonate membranes to diethylzinc (Zn(C_2_H_5_)_2_ or DEZn)) and H_2_O vapor, separately by N_2_ purge at a flow rate of 100 sccm at 80 °C. The ALD process consisted of 0.1 s pulse of DEZn, 20 s of exposure of the cyclopore polycarbonate membranes to DEZn, 40 s of N_2_ purge followed by a 2 s pulse of H_2_O, 30 s exposure to H_2_O, and a final 60 s N_2_ purge. After 250 ALD cycles, ZnO films with 50 nm ZnO thickness were deposited on the cyclopore polycarbonate membranes. To finally remove the membrane template, heat treatment at 450 °C for 4 h was performed under ambient atmosphere. Figure 1 shows a schematic illustration of the process for fabricating the hollow ZnO NWs.

### Characterization

The morphology of the synthesized hollow ZnO NWs was studied by field emission scanning electron microscopy (FE-SEM, S-4300SE, Hitachi). The phase and crystallinity were examined by X-ray diffraction (XRD, X’pert MPD PRO, Philips), and the specific surface areas were measured by Brunauer–Emmett–Teller (BET) analysis.

### Gas sensing test

The process for fabrication of the sensors is described in detail in our previous publications [17, 18]. We applied the interdigitated electrode on the surface of the sensing layer deposited on the substrate. In other words, the interdigitated electrode was made on top of the sensing layer by sputtering with a metal shadow mask. For the interdigitated electrode, Ti (~ 50 nm in thickness) and Pt (~ 150 nm) double layers were sequentially deposited on the sensing layer via sputtering using an interdigital electrode shadow mask.

The sensing properties of the hollow ZnO NW sensors were investigated in the presence of CO (reducing gas) and NO_2_ (oxidizing gas). The sensing measurements were performed at different temperatures using a home-made gas dilution and testing system. To avoid any possible variation in the sensing properties, the gas concentration was controlled by changing the mixing ratio of the dry air-balanced target gas and dry air through accurate mass flow controllers, with total flow rate of 500 sccm. The response of the fabricated sensors was determined as follows:

1 $$ R \, = \, {{R_{a} } \mathord{\left/ {\vphantom {{R_{a} } {R_{g} }}} \right. \kern-0pt} {R_{g} }}\quad {\text{For CO gas}} $$

2 $$ R \, = {{R_{g} } \mathord{\left/ {\vphantom {{R_{g} } {R_{a} }}} \right. \kern-0pt} {R_{a} }}\quad {\text{For NO}}_{ 2} \;{\text{gas}} $$

where *R*_*a*_ and *R*_*g*_ are the resistances in the absence and presence of the target gas, respectively.

## Results and discussion

### Structural and morphological study

**Fig. 2:**
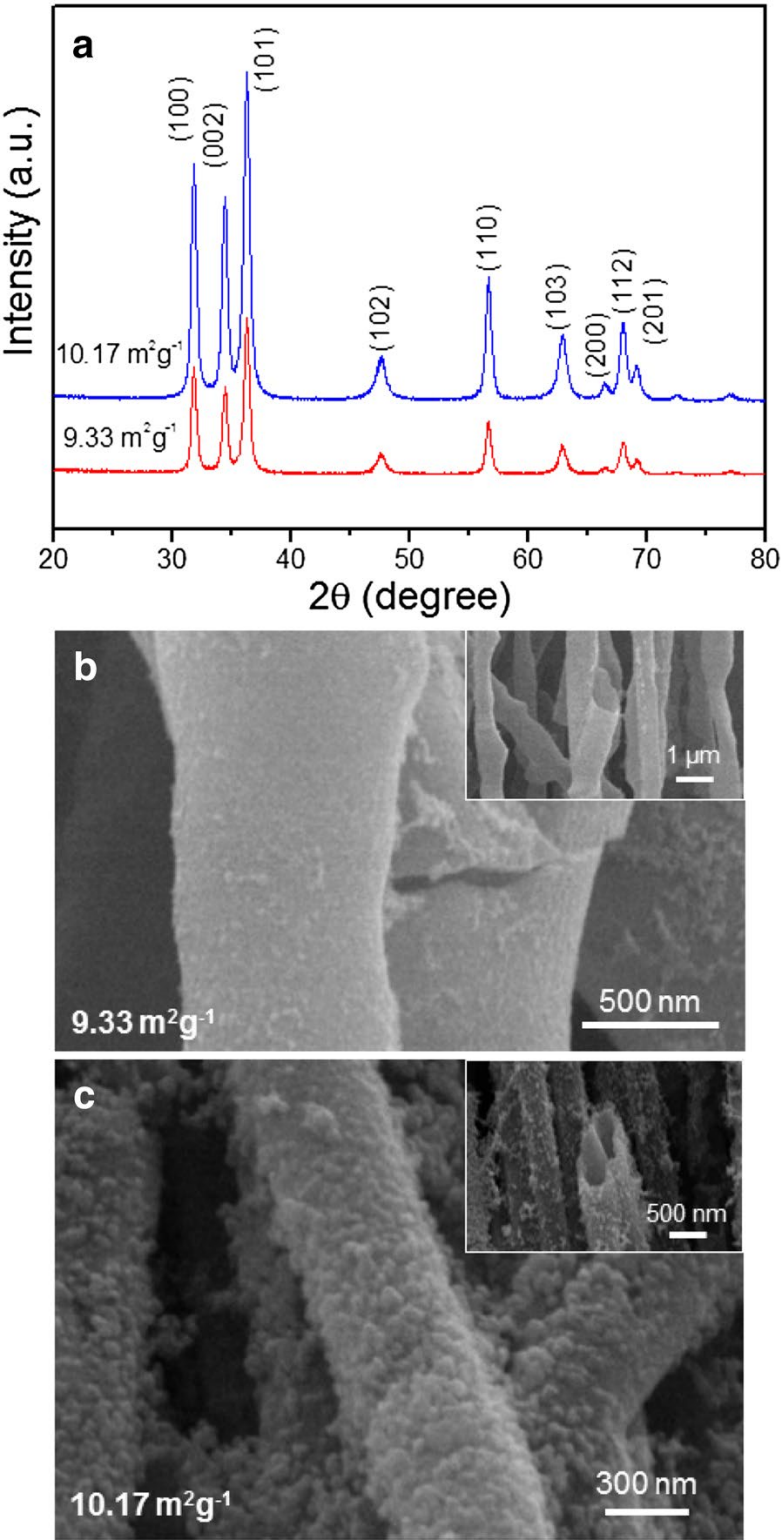
**a** XRD patterns of hollow ZnO NWs. FE-SEM images of hollow ZnO NWs with different surface areas: **b** 9.33 m^2^ g^−1^. **c** 10.17 m^2^ g^−1^

The XRD patterns of the hollow ZnO NWs are shown in Fig. 2. The diffraction peaks at 2θ values of 31.72°, 34.38°, 36.26°, 47.61°, 56.63°, 62.90°, 66.35°, 67.98°, and 69.11° could be indexed to the (100), (002), (101), (102), (110), (103), (200), (112), and (201) lattice planes of ZnO with the wurtzite hexagonal crystal structure (JCPDS Card No. 36-1451). No other diffraction peaks were observed in the XRD patterns, indicating successful removal of the polycarbonate membrane templates upon heat treatment at 450 °C.

The morphologies of the hollow ZnO NWs were observed by FE-SEM. Figure 2a, b show typical FE-SEM images of the hollow ZnO NWs with different surface areas (i.e., 9.33 and 10.17 m^2^ g^−1^) prepared from the membranes with pore sizes of 1 and 0.4 µm, respectively. As shown in Fig. 2a, the hollow ZnO NWs synthesized using the membrane with a pore diameter of 1 µm had a relatively smooth surface morphology. The inset in this figure clearly shows the hollow nature of the synthesized ZnO NWs. However, the surfaces of the hollow ZnO NWs prepared using the membrane with a pore diameter of 0.4 µm had bead-like humps, which obviously increased the surface area of this sample. The inset in this figure again shows the hollow nature of the ZnO NWs.

### Gas sensing study

**Fig. 3:**
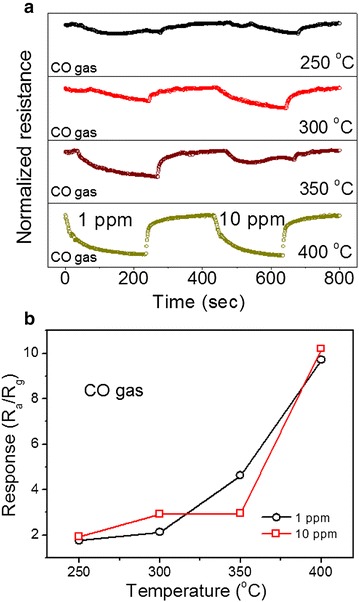
**a** Normalized resistance curves of hollow ZnO NW sensor with surface area of 10.17 m^2^ g^−1^ towards 1 and 10 ppm CO gas at different temperatures. **b** Corresponding response versus temperature plots for 0.1 and 1 ppm CO gas

The temperature is one of the most important parameters affecting the sensing behavior of a sensor. This is because of the fact that the adsorption, reaction, and desorption phenomena are strongly dependent on the temperature [19]. In order to determine the optimal working temperature, the higher surface area (10.17 m^2^ g^−1^) gas sensor was exposed to 1 and 10 ppm of CO gas at different temperatures. Figure 3a shows the normalized resistance curves of the sensor for 1 and 10 ppm of CO over the temperature range of 250–400 °C. Notably, the resistance of the sensor decreased when CO gas was supplied and increased when the supply was discontinued. This clearly indicates n-type behavior of the gas sensor, originating from the n-type nature of ZnO as a result of oxygen defects in the structure of ZnO. At 250 °C, there was no noticeable response to CO gas. At 300 and 350 °C, even though the response was higher than that at 250 °C, the response was still not significant. However, at 400 °C, a significant response was observed. Therefore, 400 °C was determined as the optimal sensing temperature. Figure 3b shows the plot of the gas response versus the sensing temperature. The low response of the gas sensor at lower temperatures is due to insufficient energy for the adsorption and reaction phenomena on the surface of the sensor.

**Fig. 4:**
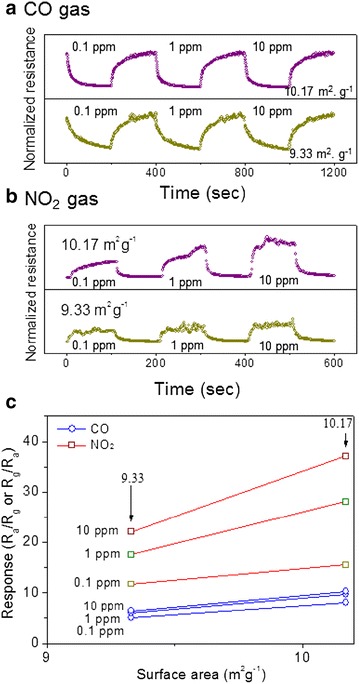
Normalized resistance curves of hollow ZnO NW sensors with different surface areas towards 0.1, 1, and 10 ppm of **a** CO and **b** NO_2_ at 400 °C. **c** Response versus surface area for hollow ZnO NWs sensor with different surface areas at different concentrations of CO and NO_2_ gases

Figure 4a, b display the dynamic normalized resistance curves of the ZnO NW sensors with different surface areas upon exposure to 0.1, 1, and 10 ppm of CO and NO_2_ gases, respectively, at 400 °C. Because NO_2_ is an oxidizing gas, the resistance of the sensors will increase upon exposure to NO_2_ gas. Figure 4c shows the response of both gas sensors versus the surface area when exposed to different concentrations of gaseous NO_2_ and CO. The response of both sensors towards NO_2_ was much higher than the response to CO gas; furthermore, the gas response increased with increasing gas concentration for both gases. Moreover, the gas sensor with the higher surface area showed a higher response to both NO_2_ and CO. Because the gas adsorption phenomenon is strongly dependent on the surface area, the sensor with the higher surface area can provide more adsorption sites for the target gases, and therefore, as expected, the sensor with the higher surface area (10.17 m^2^ g^−1^) showed better sensing performance than the sensor with the lower surface area (9.33 m^2^ g^−1^).

The gas sensing mechanism of metal-oxide based gas sensors is based on a change in the resistance upon gas adsorption and desorption. In air, oxygen gas will be adsorbed on the surface of the sensor, and owing to its high electron affinity, oxygen can extract electrons from the conduction band of ZnO to form various oxygen ions according to the following reactions:

3 $$ O_{ 2} \left( g \right) \to O_{ 2} \left( {ads} \right) $$

4 $$ O_{ 2} \left( {ads} \right) + e^{ - } \to O_{ 2}^{ - } $$

5 $$ O_{2}^{ - } \left( {ads} \right) + e^{ - } \to 2O^{ - } $$

6 $$ O_{ 2} \left( {ads} \right) + e^{ - } \to O^{2 - } $$

**Fig. 5:**
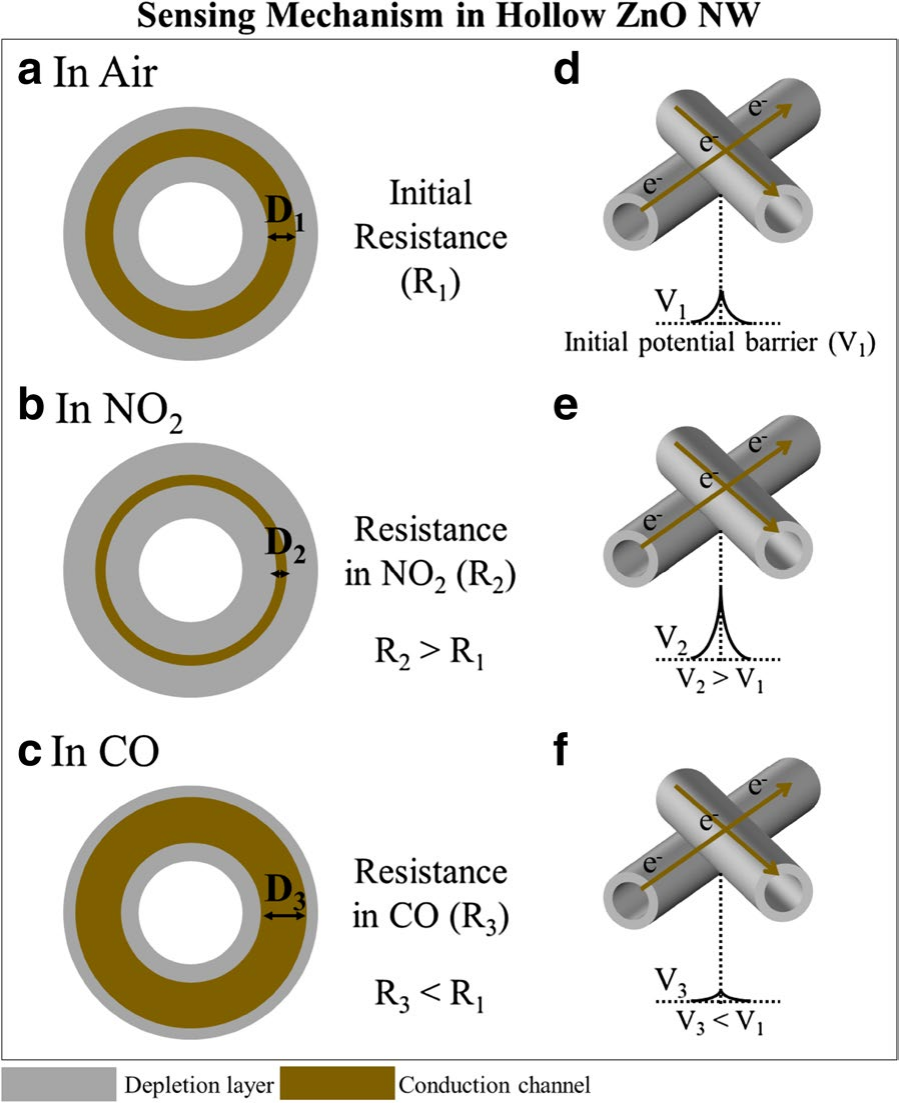
Schematic illustration of sensing mechanism in hollow ZnO NWs. Changes in depletion layers in **a** air, **b** NO_2_, and **c** CO. Changes in potential barriers in **d** air, **e** NO_2_, and **f** CO gas

It is reported that the $O_{2}^{ - }$, *O* ^−^, and *O* ^2−^ ions are respectively stable at < 150, 150–300, and > 300 °C [20, 21]. Herein, the sensing temperature was 400 °C; thus, it can be reasonably supposed that the dominant oxygen species on the surface of the sensor was *O* ^2−^. Abstraction of electrons from the surface of ZnO by oxygen leads to the formation of an electron depleted layer (EDL) on the inner and outer surfaces of the sensor, and the width of the conduction channel is proposed to be D_1_, as shown in Fig. 5a.

When the ZnO sensor is exposed to NO_2_ gas, NO_2_ can directly take electrons from the surface of the sensor or can react with the adsorbed oxygen species on the surface of the sensor [21] as follows [22, 23]:

7 $$ NO_{ 2} + e^{ - } \to NO_{ 2}^ {-} $$

8 $$ NO_{2}^{ - } + O^{ 2- } + e^{ - } \to NO\left( g \right) + 2O^{ 2-} $$

These reactions will result in a decrease in the electron concentration and an increase in the width of the depletion layer, and an increase in the resistance (see Fig. 5b). Accordingly, the width of the conduction channel decreases to D_2_, which is smaller than D_1_ (in air), and a high response can be observed.

Upon exposure of the sensor to CO gas, the gas reacts with adsorbed electrons on the surface of the sensor according to the following reaction [24]:

9 $$ CO + O^{2 - } \to CO \left( {gas} \right) + 2e^{ - } $$

The released electrons return to the surface of the ZnO sensor, increasing the width of the depletion layers on the inner and outer surfaces of ZnO; the width of the conduction channel will increase to D_3_, which is larger than D_1_. Accordingly, the resistance will decrease (see Fig. 5c). For ZnO NWs-based gas sensors, the modulation of depletion layers in the presence of target gas, has been reported in many papers. For instance, Choi et al. [25] reported modulation of depletion layers in the networked ZnO NWs in the presence of CO gas. Drobek et al. [26] reported modulation of depletion layers in pristine ZnO and ZnO@ZIF-8 composite NWs in the presence of some reducing gases. Additionally, for other metal oxide NWs such as SnO_2_ NWs [27] and In_2_O_3_ NWs [28], the same sensing mechanism has been proposed.

Resistance modulation may also arise from homojunctions formed as a result of intersections between the hollow ZnO NFs. As shown in Fig. 5d–f, when the sensor is exposed to NO_2_ gas, the initial potential barrier in V_1_ will increase to V_2_, and upon exposure to CO gas, it decreases to V_3_, which is lower than V1. These resistance modulations eventually contribute to observation of a response in the sensors.

The higher response to NO_2_ relative to CO may be related to the high electron affinity of NO_2_ (2.28 eV) in comparison with that of adsorbed oxygen (0.43 eV) [29]. NO_2_ is a strongly oxidizing gas that can extract electrons from the exposed surfaces of the hollow ZnO NFs and significantly decreases the width of the electron depletion layers.

Table 1 presents a comparison of some ZnO-based gas sensors for the detection of NO_2_ gas with that of the present hollow ZnO NWs sensor. As is evident, the developed sensor based on membrane template-grown ZnO NWs shows a better response towards NO_2_. In particular, the developed sensor showed a response of 15.5 to 0.1 ppm NO_2_, whereas the response of branched ZnO NWs to 5 ppm was 1.06 [19]. Further, the response of the ZnO nanoparticles towards 1 ppm NO_2_ was 13.7 [30]. This high response observed in the present hollow ZnO NWs sensor can be mainly attributed to the high surface area of the synthesized hollow ZnO NFs, where the inner and outer surfaces both provide numerous adsorption sites for NO_2_ gas. One paper [40] listed in Table 1 reports the value of specific surface area of sensor materials. Flower-like ZnO revealed a specific surface area of 4.9 m^2^ g^−1^, supporting the high surface area of the hollow ZnO NFs. However, the sensing temperature used herein is relatively high in comparison with those of the other sensors.

**Table 1:** Comparison of the NO_2_ gas sensing properties of the present sensor (with specific surface area of 10.17 m^2^ g^−1^) with those of other ZnO-based gas sensors reported in the literature

Sensor	NO_2_ conc. (ppm)	*T* (°C)	Response (*R*_*a*_/*R*_*g*_)	References
Hollow ZnO NWs	0.1	400	15.5	This study
Hollow ZnO NWs	10	400	37.1	This study
Branched ZnO NWs	5	300	1.06	[19]
ZnO-decorated MWCNTs	10	300	1.023	[20]
ZnO/graphene nanocomposites	1	300	12.57	[21]
CNT-ZnO nanocomposite	20	250	1.19	[31]
SnO_2_-core/ZnO-shell NFs	5	300	1.5	[32]
SnO_2_–ZnO–Co NWs	10	300	7.48	[33]
Zn_2_SnO_4_/ZnO nanorods	1	300	1.70	[34]
ZnO brushes	50	300	1.2	[35]
ZnGa_2_O_4_-core/ZnO-shell NWs	1	250	2.6	[36]
ZnO nanoparticles	1	150	13.7	[30]
ZnO nanorods	50	225	35	[37]
ZnO nanorods	5	175	20	[38]
ZnO-reduced graphene oxide	5	25	2.5	[39]
Flower-like ZnO (4.9 m^2^ g^−1^)	100	25	12.27	[40]

## Conclusion

In summary, a novel approach was applied to the fabrication of hollow ZnO NWs with different specific surface areas. Cyclopore polycarbonate membranes with different pore sizes were used as templates and ZnO was deposited on these templates via the ALD technique. Because of the simplicity of this method, it can be easily applied to other oxide semiconductors. The prepared hollow ZnO NWs had respective surface areas of 9.33 and 10.17 m^2^ g^−1^. Gas sensors were fabricated from the hollow ZnO NWs, and the gas sensing properties were investigated in the presence of CO and NO_2_ gases. The sensor with a surface area of 10.17 m^2^ g^−1^ showed excellent sensing of NO_2_ at 400 °C relative to the lower surface area gas sensor; the responses to 0.1, 1, and 10 ppm NO_2_ were 15.5, 28.06, and 37.1, respectively.
